# Bacterial species identification using MALDI-TOF mass spectrometry and machine learning techniques: A large-scale benchmarking study

**DOI:** 10.1016/j.csbj.2021.11.004

**Published:** 2021-11-09

**Authors:** Thomas Mortier, Anneleen D. Wieme, Peter Vandamme, Willem Waegeman

**Affiliations:** a KERMIT, Department of Data Analysis and Mathematical Modelling, Faculty of Bioscience Engineering, Ghent University, Coupure links 653, B-9000 Ghent, Belgium; b BCCM/LMG Bacteria Collection, Laboratory of Microbiology, Faculty of Sciences, Ghent University, K.L. Ledeganckstraat 35, B-9000 Ghent, Belgium

**Keywords:** Bacterial species identification, MALDI-TOF MS, Machine learning, Extreme classification, Hierarchical classification, Neural networks

## Abstract

Today machine learning methods are commonly deployed for bacterial species identification using MALDI-TOF mass spectrometry data. However, most of the studies reported in literature only consider very traditional machine learning methods on small datasets that contain a limited number of species. In this paper we present benchmarking results on an unprecedented scale for a wide range of machine learning methods, using datasets that contain almost 100,000 spectra and more than 1000 different species. The size and the diversity of the data allow to compare three important identification scenarios that are often not distinguished in literature, i.e., identification for novel biological replicates, novel strains and novel species that are not present in the training data. The results demonstrate that in all three scenarios acceptable identification rates are obtained, but the numbers are typically lower than those reported in studies with a more limited analysis. Using hierarchical classification methods, we also demonstrate that taxonomic information is in general not well preserved in MALDI-TOF mass spectrometry data. For the novel species scenario, we apply for the first time neural networks with Monte Carlo dropout, which have shown to be successful in other domains, such as computer vision, for the detection of novel species.

## Graphical abstract

**Figure:**
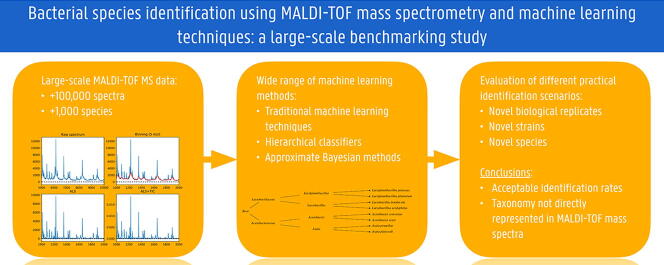

## 1 Introduction

In the last decade, matrix-assisted laser desorption/ionization time-of-flight mass spectrometry (MALDI-TOF MS) has emerged as a novel identification method for a wide range of bacterial species. Compared to sequencing a bacterial genome, it is a cheaper and faster alternative for situations where a lot of strains need to be analyzed on a daily basis [1], [2]. Today, MALDI-TOF MS is routinely used in clinical laboratories for the identification of pathogenic strains, but it is also increasingly applied to identify environmental and food-related microbiota [3], [4], [5], [6], [7], [8], [9], [10]. MALDI-TOF MS generates mass spectra that quantify mostly ribosomal proteins and peptides present in a purified culture. As these proteins are highly specific for a bacterial species, mass spectra can be seen as species-specific fingerprints, allowing for an accurate identification of purified strains at the genus and species level [11], [12].

The analysis of a sample typically starts with isolating a set of strains using different cultivation conditions, followed by a purification step, in which biomass is amplified to a sufficient level [13], [14], [15]. After cultivation, which is the most time-consuming part of the analysis, microbial cells or crude extraction thereof are deposited onto a metal target plate and subsequently overlaid or mixed with an appropriate organic matrix solution. As a last step, the unlabeled spectrum of a novel strain is identified using software tools, which typically consist of one or several machine learning algorithms as most important building blocks. In the present work we focus on this last step of the identification process.

In a recent survey, Weis et al. [16] discussed 36 studies that deployed machine learning algorithms for bacterial species identification and antimicrobial susceptibility testing using MALDI-TOF MS. Remarkably, the vast majority of these studies apply off-the-shelf classification methods in combination with relatively-small datasets, typically containing less than a thousand samples and a very small number of species, which are often limited to a single family or even a single genus. This can be attributed to the specific interest of individual research labs, and the substantial labour cost of collecting a large dataset. Yet, also the business model of MALDI-TOF MS manufacturers such as Bruker Daltonik GmbH & Co. KG (Germany, http://www.bruker.com/) and bioMérieux (France, http://www.biomerieux.com/) plays an important role. In addition to mass spectrometers, those vendors sell software packages that have access to private databases with annotated spectra [17]. As soon as research labs bring a new dataset in the public domain, this dataset is acquired by industrial players, and appended to their commercial databases. As a result, commercial software slowly becomes the gold standard, but the black-box nature of this software poses serious threats w.r.t. transparency, reliability and reproducibility.

The goal of the present paper is to provide an independent evaluation of machine learning methods for bacterial species identification with MALDI-TOF MS. To this end, we consider three different identification scenarios that are of practical relevance.

In a first scenario, we assume that at test time only novel biological replicates of strains seen during training are analyzed, and one therefore expects a good identification performance.

In a second and perhaps more realistic scenario, where also novel strains of previously-encountered species need to be identified at test time, one can expect a drop in performance compared to the first scenario. In a third scenario, for which specific methods are required, we investigated how well novel species can be detected at test time. For those three scenarios we make the following contributions:

1. For the first two scenarios, we present benchmarking results on an unprecedented scale, using several datasets that contain, together, almost 100,000 spectra from strains that represent more than 1000 species. We experiment with classical machine learning algorithms and more recent classification methods, such as deep learning and hierarchical classification methods. These last two groups of methods only work well for large-scale datasets and have not yet been applied yet to MALDI-TOF MS data.
2. The hierarchical classification methods that we implemented utilize phylogenetic information in the form of a taxonomic hierarchy. By comparing the identification performance of such methods with the performance of methods that ignore phylogenetic information, we also investigate to what extent evolutionary relationships among species are preserved in MALDI-TOF MS data.
3. For the novel species scenario, the goal is to detect whether a MALDI-TOF MS sample represents a species that is not present in the training dataset. In this scenario, which inherently translates to an out-of-distribution detection task (i.e., binary classification), the traditional classification methods in previous scenarios are not directly applicable. We present promising results with dropout-based neural networks, which have recently become very popular in areas like computer vision [18], [19].
4. For the novel species scenario, the goal is to detect whether a MALDI-TOF MS sample represents a species that is not present in the training dataset. In this scenario it is not possible to apply off-the-shelf classification methods.

The structure of the present manuscript is straightforward. In Section 2, we give an overview of the datasets and experiments that are considered in this work. We also discuss in detail the preprocessing and machine learning methods that are used to address the above research questions. In Section 3, we present numerical results for the three different scenarios. In Section 4, we discuss the implications of these results for the community, and we make a connection with previous studies. In Section 5, a short conclusion is formulated.

## 2 Materials and methods

### 2.1 Datasets

In order to address the above research questions, we will work with three datasets, referred to as the global dataset (GD), lyopreservation dataset (LYO) and additional test dataset (TEST). In what follows we provide information on how the three datasets were generated.

The global dataset is a historical database that contains samples for more than 2400 taxonomic reference strains, representing more than 1000 bacterial species, cultured and analyzed in the last decade by researchers in the Laboratory for Microbiology LM-UGent (Ghent University, Belgium). Strains are considered reference strains in bacterial taxonomy when they are chosen as type specimen during the formal description and naming of novel bacterial species, or when they have been included in comprehensive taxonomic studies [20]. The reference strains included in the global dataset were identified using state-of-the-art identification methods available at the time of isolation and subsequently stored in the BCCM/LMG Bacteria Collection. More precisely, for the recent deposits in the BCCM/LMG Bacteria Collection, strain information was acquired by means of whole genome sequencing. Yet, for the historically-deposited strains, other techniques were used for identification, such as DNA-DNA hybridization or housekeeping gene sequence analysis.

All strains were cultivated according to the provider’s instructions to a third generation. Afterwards, bacterial cell extracts (1 μl) were spotted seven to eight times on a target plate (Bruker Daltonik GmbH & Co. KG, Germany) and dried in air at room temperature. The sample spot was overlaid with 1 μl of matrix solution (10 mg/ml α-cyano-4-hydroxycinnamic acid in acetonitrile–water-trifluoroacetic acid [TFA] [50:47.5:2.5]). Each target plate comprised one spot of pure matrix solution, used as a negative control, and one spot of Bacterial Test Standard (Bruker Daltonik GmbH & Co. KG, Germany), used for calibration. The target plate was measured automatically for four times on a Bruker Microflex LT/SH (Smart) system (Bruker Daltonik GmbH & Co. KG, Germany), thus obtaining a total of 28 to 32 technical replicate spectra for each strain. The spectra were obtained in linear, positive ion mode using FlexControl (v3.4) software according to the manufacturer’s recommended settings (Bruker Daltonik GmbH & Co. KG, Germany). Each final spectrum resulted from the sum of the spectra generated at random positions to a maximum of 240 shots per spectrum.

The lyopreservation dataset is a small dataset that was released in 2019 [21]. This dataset can be subdivided into two subsets, further denoted as LYO1 and LYO2. Both subsets contain the same strains, but MALDI-TOF mass spectra were generated before and after lyophilization and subcultivation of the strains. This makes this dataset ideally suited to evaluate the performance of MALDI-TOF MS for the scenario where novel biological replicates of previously-encountered strains need to be identified. All samples of the lyopreservation dataset were analyzed using the same protocol as the global dataset.

The additional dataset TEST contains MALDI-TOF mass spectra generated from isolates obtained in a recent microbial diversity project, in which the bacterial and yeast fraction of a food sample was studied. MALDI-TOF MS data acquisition was performed as described above for the global dataset GD, except that microbial extracts were spotted in duplicate and measured only once. For this dataset, ground-truth species labels were assigned based on the highest match obtained after matching the mass spectra generated with those present in the BDAL (Bruker Daltonik GmbH & Co. KG, Germany) and the LM-UGent in–house identification databases. A fraction of mass spectra that did not match with a Bruker logscore higher than 1.8 were left out from the final dataset. This dataset contains mass spectra generated from isolates of species that are not represented in the global dataset GD. Some isolates were classified to *Pichiaceae*, *Saccharomycodaceae* and *Cellulomonadaceae*, three families that are not represented in the global dataset GD. Others belonged to species that are not present in the global dataset yet classified as *Acetobacteraceae*, a family that occurs frequently in the global dataset GD.

### 2.2 Data preprocessing

In what follows, let us denote a dataset by $D={\left\{(S_{i},y_{i})\right\}}_{i=1,\ldots ,N}$, consisting of *N* MALDI-TOF mass spectra, in which the *i*-th sample $S_{i}$ has species label $y_{i}$. A MALDI-TOF mass spectrum.

of length $L_{i}$. is formally represented as a set $S_{i}={\left\{(x_{j},\lambda_{j})\right\}}_{j=1,\ldots ,L_{i}}$, with $\lambda_{j}$ the measured intensity value for a given m/z ratio $x_{j}$. With this notation we emphasize that different spectra may vary in length.

Most of the machine learning methods described in literature, and more precisely the ones that are going to be considered in this work, require MALDI-TOF mass spectra with a fixed-length representation as input. As such, the first preprocessing step to be considered is the so-called binning step. This step consists of (i) dividing the m/z dimension in intervals (or bins), and (ii) aggregating intensity values in each obtained interval, for each spectrum $S_{i}$. For aggregation, we choose to work with the maximum of intensities, as this is frequently used in literature [22]. The next steps in our preprocessing pipeline are the following transformations: (i) baseline correction, where an estimated baseline in each spectrum is removed by using the asymmetric least squares method (ALS) and (ii) total ion current normalization (TIC), where each intensity $\lambda_{\mathrm{ij}}$ is transformed by means of

$${\overset{\sim }{\lambda }}_{\mathrm{ij}}=\frac{\lambda_{\mathrm{ij}}}{\overset{L_{i}}{\underset{j\prime =1}{\sum }}\lambda_{\mathrm{ij}\prime }},$$

such that the sum of intensities for every spectrum $S_{i}$ sums to one [23], [24], [25], [26].

### 2.3 Flat classification methods

In general, bacterial identification can be translated to a multi-class classification problem, where the goal is to assign the correct class, i.e. taxonomic label such as strain, species, genus or family, to a given spectrum. More formally, let’s assume that the given dataset D is drawn from a distribution P(S,c) on X×Y, with X the instance space consisting of spectra and $Y=\{c_{1},\ldots ,c_{K}\}$ a class space consisting of *K* classes. Furthermore, we will assume probabilistic multi-class classifiers, which estimate conditional class probabilities P(·∣S) over Y, with properties $\forall c\in Y:0⩽P(c\;|\;S)⩽1,\sum_{c\in Y}P(c\;|\;S)=1$.

Bearing in mind the availability of taxonomic information, the corresponding classification problem w.r.t. Y can be solved by using flat or hierarchical classification models. With a flat classification model one typically denotes a model that ignores hierarchical information. In contrast, a hierarchical classification model will utilize hierarchical information for classification purposes. In the experiments, we analyzed the following flat classifiers, which can be applied to a large-scale classification setting: random forests (RF), logistic regression (LR), support vector classifier with linear kernel (LSVC), k-nearest neighbours (KNN) and a one-dimensional convolutional neural network (1DCNN), which consists of one-dimensional convolutional, batch normalization and max-pool layers. Details on how those methods were configured are given below.

In principle, one can generate conditional class probabilities with all the methods that are considered. However, for certain methods, such as random forests and k-nearest neighbors, those probabilities may not be well-calibrated. At test time, for a novel sample *S* a species name *y* is assigned by returning the mode of the conditional class distribution:

$$y=\arg\underset{c\in Y}{\max}P(c\;|\;S)\text{.}$$

Since only the mode of the conditional class distribution is needed, the probabilities themselves do not need to be well-calibrated.

### 2.4 Hierarchical classification methods

**Fig. 1:**
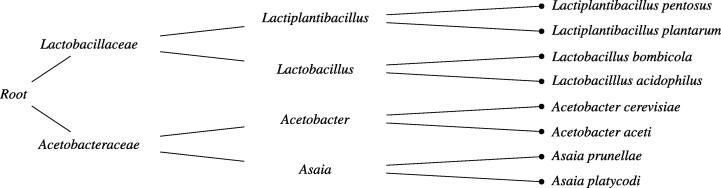
Example of a phylogenetic tree for eight bacterial species, starting from the root node, which consists of three phylogenetic levels: family, genus and species.

In order to implement hierarchical classification models, we obtained a phylogenetic tree from the NCBI Taxonomy Database [27], [28]. We ran experiments with the local classifier per parent node (LCPN) approach [29]. This approach underlies many popular algorithms such as nested dichotomies [30], [31], [32], conditional probability estimation trees [33], probabilistic classifier trees [34], or hierarchical softmax [35], often used in neural networks as an output layer. In a basic implementation, a multi-class classifier is trained in each parent node (i.e., all nodes except the leaves) within the taxonomy. For example, for the taxonomy represented as a binary tree in Fig. 1, one would need to train seven separate base learners. Each base learner is trained to distinguish between its child nodes. When probabilistic classifiers are used as base learners, a hierarchical factorization of the conditional class distribution P(c∣S) is learned, where one can express the conditional class probability for a particular leaf node via the chain rule of probability:

$$P(c\;|\;S)=\underset{v\in \mathrm{Path}(c)}{\prod }P(v\;|\;\mathrm{Parent}(v),S),$$

where Path(c) is a set of nodes on the path connecting the leaf and the root of the tree structure. Parent(v) gives the parent of node *v*, and for the root node *r* we have P(r∣Parent(r),S)=1.

In the experiments, we implemented the same base learners as used for flat classification, i.e., random forests (RF), logistic regression (LR), support vector classifier with linear kernel (LSVC), k-nearest neighbours (KNN) and a one-dimensional convolutional neural network (1DCNN). At test time, a top-down approach is used to determine the most likely species label: starting from the root node, the child node with highest probability is chosen and this process is repeated until a leaf node is reached. Every leaf node corresponds to a species.

### 2.5 Novel species detection methods

As discussed above, we consider the scenario where new species, not yet seen during training, may arrive during test time. In machine learning jargon, the corresponding task is often referred to as out-of-distribution detection [36], [37], [38], [39], open-set recognition [40], [41], [42] or anomaly detection [43]. Many novel methods for such tasks have been introduced recently, but none of those methods have been applied to bacterial species identification yet. In this work, we ran experiments with neural networks and the Monte Carlo dropout method [18]. In this method, dropout is applied both during the training and testing of neural networks and can be seen as an approximation to exact Bayesian inference in Bayesian neural networks. In contrast to neural networks, where point estimates of the weights are learned by maximizing the likelihood of the data, Bayesian neural networks are trained by learning distributions over weights [44]. By using approximate Bayesian inference, the aim is to detect novel species by analyzing the total uncertainty in predictions. Moreover, in exact Bayesian inference and given a new spectrum $S^{*}$, one can measure the total uncertainty in a prediction by means of the Shannon entropy of the posterior predictive distribution [45]:

(1) $$H[P(\cdot \;|\;S^{*},D)]=-\underset{c\in Y}{\sum }P(c\;|\;S^{*},D)\log_{2}P(c\;|\;S^{*},D)\text{.}$$

The posterior predictive distribution can then be written as follows:

(2) $$P(c\;|\;S^{*},D)=\int_{W}P(c\;|\;w,S^{*})P(w\;|\;D)dw,$$

with the posterior distribution over the weights given by Bayes’ rule:

(3) $$P(w\;|\;D)=\frac{P(D\;|\;w)P(w)}{\int_{W}P(D\;|\;w)P(w)dw},$$

and P(D∣w) the likelihood of the data. Note that in the above notations, a probabilistic classifier is parametrized by a vector of weights w. Unfortunately, calculating Eq. 2 is intractable due to the integration over the weight space W and the calculation of the posterior distribution P(w∣D), which in turn is also intractable due to the denumerator in Eq. 3. However, one can approximate the posterior predictive distribution by a finite ensemble $\left\{w_{1},\ldots ,w_{M}\right\}$, by means of learning different classifiers on *M* boostrap samples or by using the Monte Carlo dropout technique in neural networks during test time [46], [18]. The posterior predictive distribution is then approximated as follows:

(4) $$P(c\;|\;S^{*},D)=\frac{1}{M}\overset{M}{\underset{i=1}{\sum }}P(c\;|\;w_{i},S^{*})\text{.}$$

Subsequently, one can approximate the total uncertainty by plugging Eq. 4 into Eq. 1:

(5) $$u_{t}(S^{*})=-\underset{c\in Y}{\sum }\left(\frac{1}{M}\overset{M}{\underset{i=1}{\sum }}P(c\;|\;w_{i},S^{*})\right)\log_{2}\left(\frac{1}{M}\overset{M}{\underset{i=1}{\sum }}P(c\;|\;w_{i},S^{*})\right)\text{.}$$

In the experiments, we use the total uncertainty of Eq. 5 to identify out-of-distribution samples, similar to [46], [47]. Ideally, in the dataset TEST, those samples should correspond to species that are not present in the dataset GD. For the Monte Carlo dropout method, we use the 1DCNN model where the number of Monte Carlo samples is set to ten (M=10). More information with respect to implementation details is given below.

### 2.6 Experimental setup and hyperparameter tuning

Different training, validation and test sets were used for the three identification scenarios that were outlined in the introduction – see Table 1 for an overview. To evaluate the novel strain identification scenario, the global dataset is split in three different datasets: ${\mathrm{GD}}_{\mathrm{train}}$, ${\mathrm{GD}}_{\mathrm{val}}$ and ${\mathrm{GD}}_{\mathrm{test}}$, in such a way that there is no overlap in terms of strains. More precisely, for each species in GD, we distribute the set of unique strains over the three datasets, such that each strain is only represented in one of the three datasets. For example, assume we have for a given species the following unique strain labels: {st.A,st.B,st.C,st.D,st.E}. After shuffling we obtain {st.D,st.C,st.A,st.E,st.B} and, subsequently, after sequentially distributing over the three datasets we have the following membership: ${\mathrm{GD}}_{\mathrm{train}}$={st.D,st.E}, ${\mathrm{GD}}_{\mathrm{val}}$={st.C,st.B} and ${\mathrm{GD}}_{\mathrm{test}}$={st.A}.

**Table 1:** Overview of the different scenarios considered in this work, together with corresponding datasets. Statistics for the datasets are shown in Table 2.

Scenario	Train	Validate	Test
Novel strains	${\mathrm{GD}}_{\mathrm{train}}$	${\mathrm{GD}}_{\mathrm{val}}$	${\mathrm{GD}}_{\mathrm{test}}$
Novel biological replicates	GD + LYO1	–	LYO2
Novel species	${\mathrm{GD}}_{\mathrm{train}}$	${\mathrm{GD}}_{\mathrm{val}}$^a^	TEST

a Only considered for early stopping during the training of 1DCNN.

**Fig. 2:**
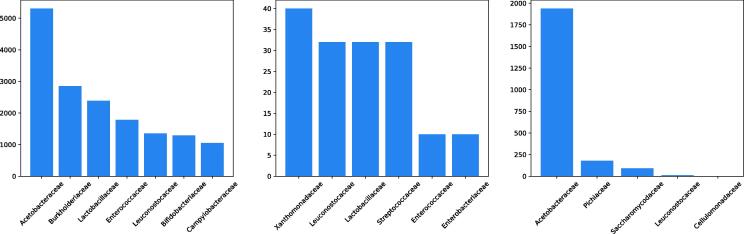
Frequency distribution for top-7 families of ${\mathrm{GD}}_{\mathrm{test}}$ (left), LYO2 (middle) and TEST (right).

Species for which only one strain has been collected, are added to the training set ${\mathrm{GD}}_{\mathrm{train}}$ in order to differentiate from the novel species scenario. ${\mathrm{GD}}_{\mathrm{train}}$ is only used for training various machine learning models, and the hyperparameters of these models are tuned on ${\mathrm{GD}}_{\mathrm{val}}$. ${\mathrm{GD}}_{\mathrm{test}}$ is used to evaluate the identification performance in the novel strains scenario. For the scenario where novel biological replicates need to be identified, the LYO2 dataset is used as test set, and the LYO1 dataset is appended to GD for training. For the novel species detection scenario, the TEST dataset is used for testing and ${\mathrm{GD}}_{\mathrm{train}}$ for training. Table 2 presents some summary statistics of the datasets and Fig. 2 shows the frequency distribution of the top-7 families for ${\mathrm{GD}}_{\mathrm{test}}$, LYO2 and TEST. One can observe a high imbalance in terms of species occurrence among the three datasets. One can see that the three datasets follow different distributions.

**Table 2:** Summary statistics for the different datasets (N – number of spectra, $K_{f}$ – number of unique families, $K_{g}$ – number of unique genera, $K_{\mathrm{sp}.}$ – number of unique species, $K_{\mathrm{st}.}$ – number of unique strains, ID – in-distribution, OOD – out-of-distribution).

Dataset	N	$K_{f}$	$K_{g}$	$K_{\mathrm{sp}.}$	$K_{\mathrm{st}.}$
${\mathrm{GD}}_{\mathrm{train}}$	50901	93	249	1087	1375
${\mathrm{GD}}_{\mathrm{val}}$	15253	24	50	211	396
${\mathrm{GD}}_{\mathrm{test}}$	21236	34	72	321	551
LYO1, LYO2	156	6	15	47	78
TEST_ID_	1950	2	5	23	833
TEST_OOD_	271	4	5	6	165

We use the novel strains experiment to test the two different preprocessing steps: binning with size 5, 10 and 20, combined with either no transformation (¬), baseline correction (ALS) or baseline correction followed by total ion current normalization (ALS + TIC). This results in nine different preprocessing combinations. Subsequently, for each of those combinations we perform hyperparameter tuning by means of randomized grid search [48], where we use five randomly sampled parameter settings for each model, on the training (${\mathrm{GD}}_{\mathrm{train}}$) and validation splits (${\mathrm{GD}}_{\mathrm{val}}$) of the global dataset. The optimal preprocessing combination and hyperparameters, obtained in the novel strains experiments, are then used throughout the other experiments.

More specifically, when it comes to hyperparameter tuning, we consider for RFs the number of trees ({50,100,150}), the number of features to consider in each split, maximum depth of the trees ({30,90,heuristic-based}), the minimum number of samples required to split an internal node ({2,5,10,15,20}) and the minimum number of samples required to be at a leaf node ({1,2,5,10,15}). For LR, we consider a linear classifier with L2 regularized cross-entropy loss and stochastic gradient descent optimization. Early stopping is applied after five iterations and the maximum number of iterations considered during optimization is set to 200 epochs. We tune the regularization strength ([0.001,…,1.0]) and learning rate ([0.001,…,1.0]). For LSVC, we consider again L2 regularization where the regularization strength is tuned ([0.001,…,1000]). For KNN we consider the number of neighbours ([1,…,20]) and the Euclidean, Manhattan and Chebyshev distance metrics.

Finally, for 1DCNN we consider two blocks consisting of a one-dimensional convolutional layer, followed by batch normalization, ReLU activation and a max-pool layer. The first block consists of a convolutional layer with eight output channels and a kernel size of ten with stride four. The second block contains a convolutional layer with 16 output channels and a kernel size of ten with stride three. For both blocks, we consider a padding and dilation of one and use kernels of size two for the max-pool layers. The fully-connected part consists of dropout, followed by one linear layer. The model is trained by optimizing the L2 regularized cross-entropy loss by means of stochastic gradient descent optimization. Early stopping is again applied after five iterations. For hyperparameter tuning, we consider the learning rate ([0.001,…,0]), weight decay ([0.001,…,0]) and dropout rate ([0,…,0.5]).

For the novel strains and novel biological replicates experiments, accuracies on all phylogenetic levels are reported. In case of flat classification, where no taxonomic information is incorporated during training, hierarchical classification is easily achieved during test time by taking the taxonomy into account. For example, a prediction delivered by a flat classifier, trained on eight different species with the taxonomy shown in Fig. 1, indirectly determines the class label on the upper genus and family level. The disadvantage of this approach is that it needs to discriminate among a large number of classes, without exploring information about parent–child class relationships present in the taxonomy.

For the novel species experiment, we consider the area under the ROC (AUROC) and precision-recall curves (AUPR), similarly as in [36], [41], [39]. All models have been implemented in Python by using the Scikit-learn and PyTorch libraries [49], [50].

## 3 Results

**Fig. 3:**
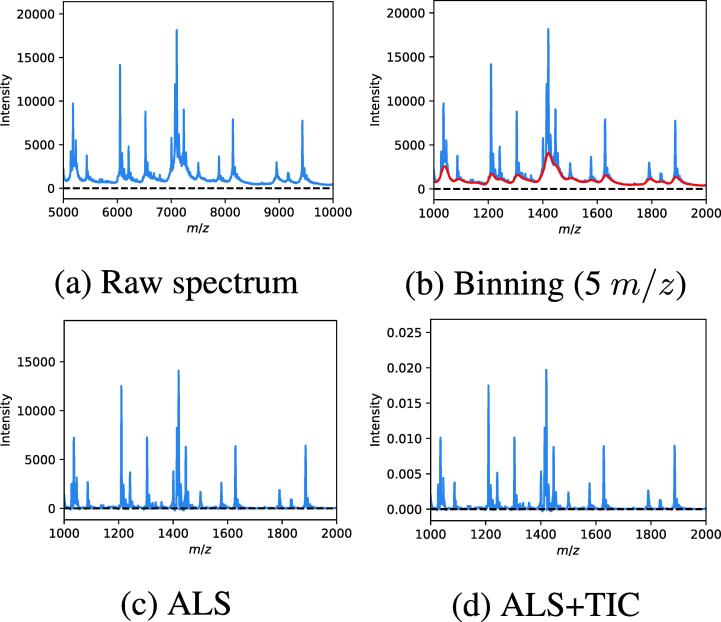
Example of a raw spectrum of Lactococcus garvieae, together with the different preprocessing steps: binning with size 5 m/z and transformation by means of baseline correction (ALS) and total ion current normalization (TIC). The estimated baseline by baseline correction is indicated by red in the top right figure. (For interpretation of the references to colour in this figure legend, the reader is referred to the web version of this article.)

In Table 3, we report the performance of different preprocessing pipelines and models in the novel strains scenario. For each model, we use the optimal set of hyperparameters obtained during randomized grid search on ${\mathrm{GD}}_{\mathrm{train},\mathrm{val}}$ and report the accuracy obtained on ${\mathrm{GD}}_{\mathrm{test}}$. In most cases, decreasing the bin size leads to a higher performance, as less information is lost during the binning process. In general, most machine learning models benefit from both transformations, except for LR, where a significant drop in performance is observed when considering total ion current normalization on top of baseline correction. A possible explanation for this result is unstable model training due to the combination of heavily reduced intensity values after TIC and the type of optimizer that is used in LR. In Fig. 3, we show a specific example of a raw spectrum of *Lactococcus garvieae*, together with all preprocessing steps considered in this work: binning and transformation by means of baseline correction and total ion current normalization. In this particular case, binning reduces the dimensionality of the original spectrum by a factor of five, while retaining most of the information present in the raw spectrum. Next, baseline correction transforms the binned spectrum by removing any baseline or trend that is present in the raw spectrum. Finally, the spectrum is rescaled such that the sum of intensities is equal to one.

**Table 3:** Hyperparameter tuning in novel strains scenario with reported accuracies (¬ – no transformation, ALS – baseline correction only, ALS + TIC – baseline correction and total ion current normalization).

Bin size	Model Transformation	LSVC	LR	RF	KNN	1DCNN
5	¬	0.7419	0.6044	0.7055	0.7321	0.8051
	ALS	0.7655	0.6626	0.7626	0.7916	0.8400
	ALS + TIC	0.8236	0.0015	0.7722	0.8478	0.8200
10	¬	0.7364	0.5430	0.7113	0.7322	0.7896
	ALS	0.7625	0.6845	0.7719	0.8069	0.8132
	ALS + TIC	0.8132	0.0015	0.7775	0.8371	0.8002
20	¬	0.7146	0.5716	0.7150	0.7260	0.7646
	ALS	0.7397	0.6815	0.7698	0.7987	0.7285
	ALS + TIC	0.8097	0.0015	0.7851	0.8263	0.7842

### 3.1 Species identification

Next, in Table 4, we present the results for the novel strains and novel biological replicates experiments. For each model, we consider a flat and hierarchical classifier and report the performance on three phylogenetic levels: family, genus and species. For all models, we consider a bin size of five and both transformations, since this combination gave rise to the best performance in the previous experiment. Only for LR, we choose to omit the total ion current normalization, given the issue raised in the previous experiment. In the novel strains scenario, KNN outperforms the other models with an accuracy of 84.78%, which brings us to the conclusion that similar spectra, i.e., with respect to some distance metric, in the input space most likely belong to the same species. When looking at the confusion matrix on family level for flat KNN in Suppl. Fig. 1, there appears to be confusion for the families *Aerococcaceae*, *Bacillaceae*, *Lactobacillaceae* and *Microbacteriaceae*. On the other hand, in the novel biological replicates scenario, the outperforming model seems to be LSVC with an accuracy of 96.15%. Again, following Suppl. Fig. 2, we observe confusion for *Lactobacillaceae* for both flat and hierarchical classifiers. In Suppl. Fig. 3, we also show the confusion matrices on genus level for both flat and hierarchical LSVC. In both scenarios, there is no gain when considering deep learning models, besides the fact that training is faster in case of large-scale classification settings. The same conclusion can be made with respect to the hierarchical classifiers, indicating that taxonomic information is not immediately represented in MALDI-TOF MS data.

**Table 4:** Results for novel strains and biological replicates scenario. Accuracies are reported on phylogenetic levels: (F) amily, (G) enus and (S) pecies. For each model, we report the performance for flat and hierarchical classification. H-XXX denotes the hierarchical classification implementation of method XXX.

Scenario	Novel strains			Novel biological replicates		
Model	${\mathrm{Acc}}_{F}$	${\mathrm{Acc}}_{G}$	${\mathrm{Acc}}_{S}$	${\mathrm{Acc}}_{F}$	${\mathrm{Acc}}_{G}$	${\mathrm{Acc}}_{S}$

LSVC	0.9494	0.9307	0.8236	0.9744	0.9615	0.9615
H-LSVC	0.9562	0.9351	0.8152	0.9423	0.9295	0.9295
LR^a^	0.7558	0.7330	0.6627	0.7179	0.6859	0.6859
H-LR^a^	0.8771	0.8446	0.7009	0.8397	0.8269	0.8141
RF	0.8851	0.8667	0.7721	0.9423	0.9103	0.8526
H-RF	0.9361	0.9221	0.8268	0.8910	0.8462	0.8141
KNN	0.9677	0.9544	0.8478	0.9744	0.9487	0.9487
H-KNN	0.9677	0.9544	0.8478	0.9744	0.9487	0.9487
1DCNN	0.9521	0.9309	0.8200	0.8846	0.8441	0.7756
H-1DCNN	0.9426	0.9110	0.7620	0.9423	0.9167	0.9038

a Total ion current normalization omitted.

### 3.2 Novel species detection

**Fig. 4:**
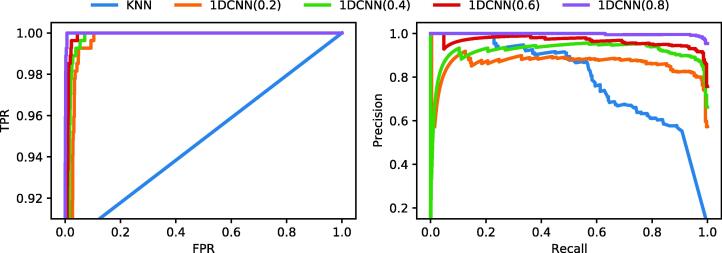
ROC and precision-recall curves obtained in the novel species scenario for KNN and 1DCNN with dropout rates of 0.2, 0.4, 0.6 and 0.8. In both plots, one observes a better performance for 1DCNN with dropout rate 0.8.

Finally, in Table 5 we evaluate whether 1DCNN with different dropout rates and KNN are useful in the context of novel species detection by using total uncertainty as defined in Eq. 5. For both KNN and 1DCNN, we use an ensemble size M=10. The other models are not considered due to the increasing training complexity of this scenario. 1DCNN with a dropout rate of 0.8 clearly outperforms KNN, considering two different performance measures: the area under the ROC curve and the area under the precision-recall curve. We also present the corresponding ROC and precision-recall curves in Fig. 4.

**Table 5:** Results for novel species scenario. Area under the ROC curve (AUROC) and area under the precision-recall curve (AUPR) are reported for out-of-distribution detection based on total uncertainty.

Model	AUROC	AUPR
KNN	0.9279	0.7934
1DCNN(0.2)	0.9882	0.8600
1DCNN(0.4)	0.9942	0.9167
1DCNN(0.6)	0.9968	0.9659
1DCNN(0.8)	0.9997	0.9975

**Fig. 5:**
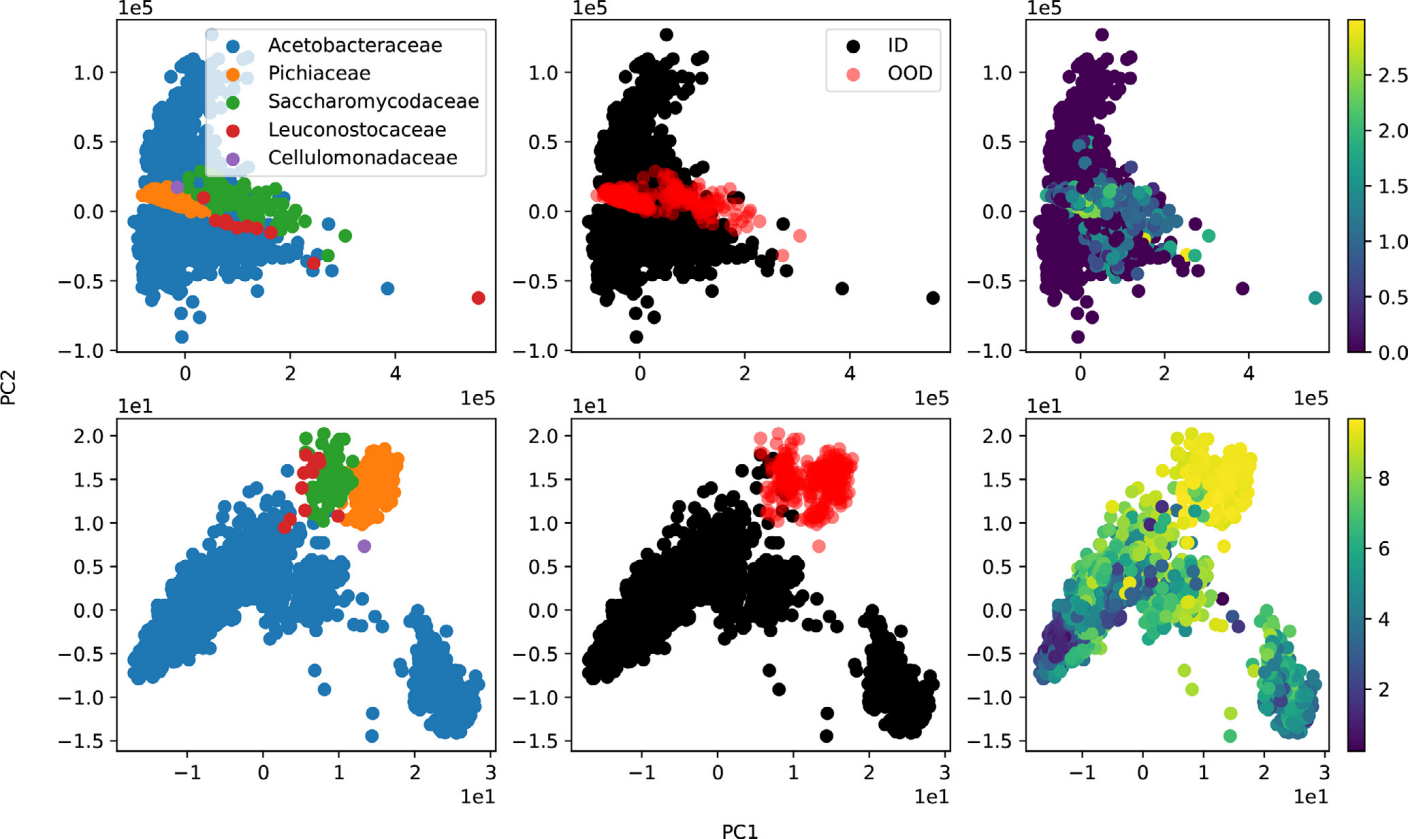
Principal component analysis applied on the original input space of dataset TEST (top) and corresponding penultimate layer of 1DCNN with dropout rate 0.8 (bottom). In all plots, the first two principal components are shown on the horizontal and vertical axis, respectively. Left color scheme: family information, center color scheme: in-distribution (ID) and out-of-distribution (OOD) information, right color scheme: estimated total uncertainty, where larger values correspond to higher total uncertainty. A better distinction between in-distribution and out-of-distribution samples is observed for the total uncertainty estimated by 1DCNN. (For interpretation of the references to colour in this figure legend, the reader is referred to the web version of this article.)

More insights into these findings are given in Fig. 5, which visualizes for the dataset TEST the first two principal components after applying principal component analysis on the original feature space and penultimate layer of 1DCNN, in the first and second row, respectively. The penultimate layer represents a learned feature representation, and in deep learning it is a common procedure to plot this space using dimensionality reduction methods such as principal component analysis. From left to right, different color schemes are applied to highlight families, in-distribution and out-of-distribution groups and the total uncertainty score of Eq. 5. One can see that out-of-distribution samples, which correspond to species not observed during training, are clearly separated in the penultimate layer of the neural network. This clear separation might be explained by the fact that the out-of-distribution samples belong to three families that are not observed during training: *Pichiaceae*, *Saccharomycodaceae* and *Cellulomonadaceae* (bottom left). In contrast, no clear separation is visible in the principal components that are derived from the original input space (top center). For the original feature space, the cluster that corresponds to out-of-distribution samples is also not characterized by a higher total uncertainty (top right), which might explain the drop in performance for KNN. Given the above findings, we conclude that novel species can be accurately identified by total uncertainty, especially when considering 1DCNN as underlying model.

## 4 Discussion

### 4.1 Size and scope of the analysis

Compared to existing papers on bacterial species identification with MALDI-TOF MS, the present paper presents benchmarking results of machine learning methods on an unprecedented scale, using a unique dataset that contains more than a thousand species, more than two thousand strains and almost 100,000 mass spectra. Weis et al. [16] discussed in a recent survey 36 studies, of which 27 conducted experiments with machine learning methods for bacterial species identification, and 9 for antimicrobial susceptibility testing. The largest of those studies considered 727 isolates [51], but many studies have less than 50 isolates – see e.g. [52], [53], [54], [55]. In addition, almost all those studies analyzed species that belong to a single family, or in some cases even a specific genus. This particular focus might be attributed to the specific interest of the research lab that collected the data, i.e., interest in clinically-relevant species, and the business model of vendors of MALDI-TOF MS machines. As soon as a new dataset is released, commercial players append their own license-based identification libraries with those datasets. License-based identification libraries form a unique selling property for commercial players, but the black-box nature of the accompanying software poses serious threats w.r.t. transparency, reliability and reproducibility.

### 4.2 Data preprocessing

In this work, the focus was less on data preprocessing. We implemented the three preprocessing steps that are mostly used in other studies: binning to obtain a fixed-length representation, and baseline correction and total ion current normalization to mitigate technical variability. As already stated, one can choose to work with equally or non-equally spaced intervals for binning, where intervals can be either overlapping or non-overlapping, but previous work has shown that all those options lead to comparable results [22]. The choice for non-overlapping equally-spaced bins is therefore mainly motivated by common practice and preservation of interpretability. Some other preprocessing steps are bundled in the open-source MALDIquant package [56]. In our opinion, those steps are mainly useful in the context of unsupervised learning, such as data visualization and clustering, but they do not influence the performance of supervised classification methods. Because of computational reasons, the recently-introduced machine learning method of Weis et al. could not be applied [57]. Inspired by topological data analysis, these authors introduced the so-called PIKE kernel. Interestingly, this is a kernel that avoids binning as a preprocessing step, and it can be used in combination with Gaussian processes and support vector machines. Unfortunately, computing the kernel matrix has a $O(N^{2}L^{2})$ complexity, making the approach unsuitable given the size of the data that we analyzed.

### 4.3 Machine learning models for species identification

For the identification of novel strains and novel biological replicates, we applied in this paper for the first time convolutional neural networks (CNNs). The other machine learning methods that we analyzed have been considered before for bacterial species identification using MALDI-TOF MS data, albeit with smaller datasets – see. e.g. [58], [59], [60], [61], [62], [63]. CNNs have become very popular recently in many fields. For large datasets with complex feature spaces, they usually outperform classical machine learning methods, since they combine the data preprocessing and learning phase in a single algorithm, while avoiding the need for domain-specific preprocessing techniques [64]. Surprisingly, our results indicate that CNNs are not able to outperform traditional methods such as KNNs and LSVMs, which yielded the best predictive performance. However, CNNs turned out to be more stable than other methods w.r.t. the application of specific preprocessing pipelines. Without preprocessing, the performance of CNNs did not drop substantially. In addition, we observed that CNNs could be trained in a very efficient manner, unlike some of the older methods, for which implementations are not always optimized to handle large datasets. A recent paper that analyzed one-dimensional CNNs for single-cell MALDI-TOF MS data came to a similar conclusion [22]. In this revolutionary technology, a spectrum is generated for every cell in a natural environment, e.g. a urine sample of a patient, while avoiding a time-consuming culturing phase. Datasets obtained in that way easily contain more than 100,000 spectra, so the need for computationally-efficient machine learning methods will probably increase in the near future.

As far as we know, this is also the first work that implements hierarchical classifiers for large-scale bacterial species identification using MALDI-TOF MS. Surprisingly, hierarchical classifiers did not outperform their flat counterparts on species level, indicating that taxonomic information is not directly represented in MALDI-TOF MS data. If spectra generated from closely related bacteria are also close w.r.t. some similarity or distance metric in the feature space, hierarchical classification is expected to be more accurate compared to flat classification [65]. This is in fact a necessary condition for improvement, as observed in fields where hierarchical classifiers have gained interest, such as text categorization [35], protein function prediction [66], [65], functional annotation of genes [67], [68], identification of plants based on images [69] and discrimination of bird songs [70]. In microbiology, hierarchical classifiers have been successfully applied to bacterial species identification using Fourier-Transform-Infrared spectra [71], [72] and fatty acid methyl esther profiles [73].

Hierarchical classifiers might be preferred over flat classifiers for other reasons than predictive performance on species level. A nice property of hierarchical classifiers is their ability to abstain at different levels in the hierarchy [29]. When there is too much uncertainty about the species name of a sample, one can decide to identify that sample only on genus or family level [74], [75]. This is nicely illustrated in our experimental results, where the accuracy on family level was in general substantially higher for hierarchical classifiers. On family level, the low performance of some flat classifiers can be attributed to the detour that these models make, i.e., they first identify a sample on species level and convert the label to family level as a post-processing step. Such a detour with a one-versus-all decomposition on species level leads to an overparameterized model when species only need to be identified on family level. As a side note, hierarchical classifiers might also be preferred over flat classifiers for computational reasons. At test time, hierarchical classifiers have logarithmic time complexity to identify samples at species level, whereas flat classifiers have a linear time complexity (w.r.t. the number of classes) [35], [76].

### 4.4 Evaluation protocols for species identification

In the present study, we analyzed three of the most common reasons why machine learning algorithms might not assign the correct species name to a MALDI-TOF mass spectrum, namely the existence of novel strains, novel biological replicates and/or novel species at test time. Remarkably, many existing studies do not make such a distinction [16]. When we compared the performance for the novel strains and novel biological replicates scenarios, we observed that the accuracy on species level increased with more than 10% if only novel biological replicates are analyzed at test time. Therefore, we believe that relatedness between spectra is indeed present at the more fine-grained strain level, since a classifier yields more accurate identifications for a particular strain when that strain also appears in the training dataset. However, it is very reasonable to test machine learning models in terms of generalization to unseen strains, as most often, novel strains may arrive during test time. Going one step further, one could also try to classify MALDI-TOF MS spectra at strain level, but then the predictive performance usually becomes too low to be useful in practice.

The most common testing protocol in literature is *k*-fold cross-validation, where, repeatedly, one of the *k*-folds is left out for testing, while the other folds are used for training. Obviously, such an approach is not able to quantify how well novel strains can be identified. Even for identification of novel biological replicates, cross-validation is a questionable approach, especially when many technical replicates are present in a dataset. Only four existing studies avoid cross-validation by evaluating models on an independent test dataset that is not used during training [77], [78], [53], [54]. In all four cases, data of one or more labs were considered for training and data of other labs for testing. This allows to make location comparisons, as MALDI-TOF mass spectra measured at different locations suffer from batch effects, which are likely to stem from differences in laboratory routine or system settings [79]. However, an independent test dataset can in principle contain a mix of novel technical replicates, novel biological replicates, novel strains, novel species and other deviations from the training data that might impact identification. So, if one intends to investigate the impact of one particular type of deviation, it is important that the test dataset is constructed in such a way that the specific source of deviation is isolated from other sources. That is in fact the main reason why we considered three different test datasets in this paper. With the dataset ${\mathrm{GD}}_{\mathrm{test}}$, we evaluated the performance for novel strains, with LYO2 we only analyzed novel biological replicates, and with the independent dataset TEST we evaluated the performance on a mix of samples, consisting of novel biological replicates, novel strains and novel species. We did not analyze the identification performance for technical replicates, because that’s practically less useful, and because previous work has studied the impact of technical variation extensively.

### 4.5 Novel species detection

For the third scenario, in which novel species had to be detected in the dataset TEST, specific machine learning methods are needed. Technically speaking, in this case, one is not solving a classification problem but an out-of-distribution detection problem. In the machine learning literature this setting is also often referred to as open-set recognition [80], [81], anomaly detection [43] or classification with reject option [76]. Inspired by applications like self-driving cars, where it is of uttermost importance to abstain from making a prediction if the uncertainty is too high, out-of-distribution detection has become a very popular research topic [82], [83], [36], [84], [85], [86]. As a result, a whole bunch of novel methods have been recently proposed, including exact and approximate Bayesian methods [87], [88], [18], models based on Dirichlet priors [38], generative models [89], [41], [90], [91], [92], [39] and distance-based methods [93], [41], [94]. Very few of these methods have been applied to bacterial species identification. Two papers identify bacterial species based on genomics data, using Naive Bayes [74] and a specific generative model [39] for detecting out-of-distribution samples. Weis et al. [57] use Gaussian processes for out-of-distribution detection on MALDI-TOF MS data, using specific preprocessing techniques [56], [95]. The commercial ClinProTool of Bruker Daltonik GmbH & Co. KG (Germany, http://www.bruker.com/) also detects out-of-distribution samples when no match in their database is found, i.e., when the similarity of the nearest neighbour drops below a certain predefined threshold.

In this work, we decided to experiment with Monte Carlo dropout, as this method is frequently used in several domains [19], [46], [83]. This type of approximate Bayesian inference seemed to work reasonably well in our setting, yet a more extensive study in which the different frameworks from literature are analysed might be desirable, and is left as potential future work. More specifically, we believe that it would be very useful to conduct experiments with methods that are able to differentiate between aleatoric and epistemic uncertainty [45]. Aleatoric uncertainty originates from inherent noise in the data, which cannot be mitigated by collecting more data, whereas epistemic uncertainty alludes to uncertainty that can be reduced if more data would be available. In fact, the total uncertainty of Eq. 5, which was considered as criterion to detect out-of-distribution samples, can be easily decomposed into an aleatoric and epistemic part. In the conducted experiments, we did not see an improvement when considering the decomposition, however, other methods might yield different conclusions.

## 5 Conclusion

In this work, we performed a large-scale benchmarking study of bacterial identification using MALDI-TOF mass spectrometry and machine learning methods. We implemented several traditional machine learning methods, as well as a few novel methods, such as one-dimensional convolutional neural networks, hierarchical classifiers and an out-of-distribution detection method. The size and the diversity of the data that we analyzed allowed us to compare three important identification scenarios that are generally not distinguished in literature, i.e., identification of novel biological replicates (i.e., isolates that represent strains that are already present in the database), novel strains (i.e., isolates that represent novel strains that belong to species that are present in the database) and novel species that are not present in the training data (i.e., isolates that represent strains of species that are not present in the database). The results demonstrate that in all three scenarios acceptable identification rates are obtained, but the numbers are typically lower than those reported in studies with a more limited analysis. Using hierarchical classification methods, we also demonstrated that taxonomic information is in general not well preserved in MALDI-TOF mass spectrometry data. For the novel species scenario, we applied for the first time neural networks with Monte Carlo dropout, which have shown to be successful in other domains, such as computer vision, for the detection of novel classes. Especially for this last scenario, we still see a lot of possibilities for benchmarking other recent methods in a separate study.

## CRediT authorship contribution statement

**Thomas Mortier:** Conceptualization, Methodology, Software, Writing-review-editing. **Anneleen D. Wieme:** Data-curation, Writing-review-editing. **Peter Vandamme:** Data-curation, Writing-review-editing, Supervision. **Willem Waegeman:** Conceptualization, Methodology, Writing-review-editing, Supervision.

## Declaration of Competing Interest

The authors declare that they have no known competing financial interests or personal relationships that could have appeared to influence the work reported in this paper.

## References

[1] Alex van Belkum, Sonia Chatellier, Victoria Girard, David Pincus, Parampal Deol, Wm Michael Dunne Jr. Progress in proteomics for clinical microbiology: MALDI-TOF MS for microbial species identification and more. Expert Rev Proteom 12(6):2015;595–605 10.1586/14789450.2015.1091731 26472137

[2] Tan K., Ellis B., Lee R., Stamper P., Zhang S., Carroll K. (2012). Prospective evaluation of a matrix-assisted laser desorption ionization–time of flight mass spectrometry system in a hospital clinical microbiology laboratory for identification of bacteria and yeasts: a bench-by-bench study for assessing the impact on time to identification and cost-effectiveness. J Clin Microbiol.

[3] Laura Ferreira, Fernando Sánchez-Juanes, Magdalena González-Ávila, David Cembrero-Fuciños, Ana Herrero-Hernández, José Manuel González-Buitrago, Juan Luis Muñoz-Bellido. Direct identification of urinary tract pathogens from urine samples by matrix-assisted laser desorption ionization-time of flight mass spectrometry. J Clin Microbiol 48(6):2010;2110–2115. 10.1128/JCM.02215-09 PMC2884468 20392910

[4] Li Wei, Sun Enhua, Wang Ying, Pan Hongwei, Zhang Yi, Li Yong, Zhang Xin, Li Chen, Lutao Du., Wang Chuanxin (2019). Rapid identification and antimicrobial susceptibility testing for urinary tract pathogens by direct analysis of urine samples using a maldi-tof ms-based combined protocol. Front Microbiol.

[5] Segawa Shunsuke, Sawai Setsu, Murata Shota, Nishimura Motoi, Beppu Minako, Sogawa Kazuyuki, Watanabe Masaharu, Satoh Mamoru, Matsutani Tomoo, Kobayashi Masayoshi, Iwadate Yasuo, Kuwabara Satoshi, Saeki Naokatsu, Nomura Fumio (2014). Direct application of MALDI-TOF mass spectrometry to cerebrospinal fluid for rapid pathogen identification in a patient with bacterial meningitis. Clin Chim Acta.

[6] Ceyssens Pieter-jan, Soetaert Karine, Timke Markus, Van den Bossche An, Sparbier Katrin, De Cremer Koen, Kostrzewa Markus, Hendrickx Marijke, Mathys Vanessa (2017). Matrix-assisted laser desorption ionization-time of flight mass spectrometry for combined species identification and drug sensitivity testing in mycobacteria. J Clin Microbiol.

[7] Wieme Anneleen D., Spitaels Freek, Aerts Maarten, De Bruyne Katrien, Van Landschoot Anita, Vandamme Peter (2014). Identification of beer-spoilage bacteria using matrix-assisted laser desorption/ionization time-of-flight mass spectrometry. Int J Food Microbiol.

[8] Marta Dušková, Ondrej Šedo, Kateřina Kšicová, Zbyněk Zdráhal, Renáta Karpíšková. Identification of lactobacilli isolated from food by genotypic methods and maldi-tof ms. Int J Food Microbiol 159(2):2012;107–114. 10.1016/j.ijfoodmicro.2012.07.029 23072695

[9] Moussa M., Cauvin E., Le Piouffle A., Lucas O., Bidault A., Paillard C., Benoit F., Thuillier B., Treilles M., Travers M.A., Garcia Céline (2021). A maldi-tof ms database for fast identification of vibrio spp. potentially pathogenic to marine mollusks. Appl Microbiol Biotechnol.

[10] Clark Andrew E., Kaleta Erin J., Arora Amit, Wolk Donna M. (2013). Matrix-assisted laser desorption ionization-time of flight mass spectrometry: a fundamental shift in the routine practice of clinical microbiology. Clin Microbiol Rev.

[11] Piseth Seng, Michel Drancourt, Frédérique Gouriet, Bernard La Scola, Pierre-Edouard Fournier, Jean Marc Rolain, Didier Raoult. Ongoing revolution in bacteriology: routine identification of bacteria by matrix-assisted laser desorption ionization time-of-flight mass spectrometry. Clin Infect Diseases 49(4):2009;543–551. 10.1086/600885 19583519

[12] Bizzini A, Jaton K, Romo D, Bille J, Prod/textquoterighthom G, Greub G. Matrix-assisted laser desorption ionization time of flight mass spectrometry as an alternative to 16S rRNA gene sequencing for identification of difficult-to-identify bacterial strains. J Clin Microbiol 49(2):2011;693–696. 10.1128/JCM.01463-10 PMC3043501 21106794

[13] Hsieh Sen-Yung, Tseng Chiao-Li, Lee Yun-Shien, Kuo An-Jing, Sun Chien-Feng, Lin Yen-Hsiu, Chen Jen-Kun (2008). Highly efficient classification and identification of human pathogenic bacteria by maldi-tof ms. Mol Cell Proteom.

[14] Food and Drug Administration. De novo request for evaluation of automatic class III designation for the VITEK MS, 2013. 510(k).

[15] Antony Croxatto, Guy Prod’hom, Gilbert Greub. Applications of MALDI-TOF mass spectrometry in clinical diagnostic microbiology. FEMS Microbiol Rev 36(2):2012;380–407 10.1111/j.1574-6976.2011.00298.x 22092265

[16] Weis Caroline (2020). Machine learning for microbial identification and antimicrobial susceptibility testing on maldi-tof mass spectra: a systematic review. Clin Microbiol Infect.

[17] Mather Cheryl A, Rivera Sheila F., Butler-Wu Susan M. (2013). Comparison of the bruker biotyper and vitek ms matrix-assisted laser desorption ionization-time of flight mass spectrometry systems for identification of mycobacteria using simplified protein extraction protocols. J Clin Microbiol.

[18] Yarin Gal, Zoubin Ghahramani. Bayesian convolutional neural networks with bernoulli approximate variational inference, 2016.

[19] Alex Kendall, Vijay Badrinarayanan, Roberto Cipolla. Bayesian segnet: model uncertainty in deep convolutional encoder-decoder architectures for scene understanding. CoRR, abs/1511.02680, 2015.

[20] Tindall B.J., Rosselló-Móra R., Busse H.-J., Ludwig W., Kämpfer P. (2010). Notes on the characterization of prokaryote strains for taxonomic purposes. Int J Syst Evol Microbiol.

[21] Charles Dumolin, Maarten Aerts, Bart Verheyde, Simon Schellaert, Tim Vandamme, Felix Van der Jeugt, Evelien De Canck, Margo Cnockaert, Anneleen D. Wieme, Ilse Cleenwerck, Jindrich Peiren, Peter Dawyndt, Peter Vandamme, Aurélien Carlier. Introducing SPeDE: high-throughput dereplication and accurate determination of microbial diversity from matrix-assisted laser desorption-ionization time of flight mass spectrometry data. mSystems 4(5):2009;1–13. 10.1128/mSystems.00437-19 PMC6739102 31506264

[22] Papagiannopoulou Christina, Parchen René, Rubbens Peter, Waegeman Willem (2020). Fast pathogen identification using single-cell matrix-assisted laser desorption/ionization-aerosol time-of-flight mass spectrometry data and deep learning methods. Anal Chem.

[23] Shixuan He, Wei Zhang, Lijuan Liu, Yu Huang, Jiming He, Wanyi Xie, Peng Wu, Chunlei Du. Baseline correction for raman spectra using an improved asymmetric least squares method. Anal Methods 6:2014;4402–4407

[24] Sauve Anne, Speed Terence (2004). Proceedings of the genomic signal processing and statistics workshop.

[25] Alejandro Cruz-Marcelo, Rudy Guerra, Marina Vannucci, Yiting Li, Ching C. Lau, Tsz-Kwong Man. Comparison of algorithms for pre-processing of seldi-tof mass spectrometry data. Bioinformatics 24(19):2008;2129–2136. 10.1093/bioinformatics/btn398 PMC2553436 18694894

[26] Yang Chao, He Zengyou, Yu Weichuan (2009). Comparison of public peak detection algorithms for maldi mass spectrometry data analysis. BMC Bioinf.

[27] Conrad Schoch, Stacy Ciufo, Carol Hotton, Sivakumar Kannan, Rogneda Khovanskaya, Detlef Leipe, Richard McVeigh, Kathleen O’Neill, Barbara Robbertse, Shobha Sharma, Vladimir Soussov, John Sullivan, Lu Sun, Sean Turner, Ilene Karsch-Mizrachi. Ncbi taxonomy: a comprehensive update on curation, resources and tools. Database 2020 10.1093/database/baaa062 PMC7408187 32761142

[28] Eric Sayers, Mark Cavanaugh, Karen Clark, James Ostell, Kim Pruitt, Ilene Karsch-Mizrachi. Genbank, 01 2019. 10.1093/nar/gky989 PMC6323954 30365038

[29] Carlos N. Silla Jr., Alex A. Freitas. A survey of hierarchical classification across different application domains. Data Min Knowl Discovery 22:2011;31–72

[30] Fox John (1997). Applied regression analysis, linear models, and related methods. Sage.

[31] Eibe Frank, Stefan Kramer. Ensembles of nested dichotomies for multi-class problems. In: Proceedings of the twenty-first international conference on machine learning, ICML ’04. ACM; 2004.

[32] Melnikov Vitalik, Hüllermeier Eyke (2018). On the effectiveness of heuristics for learning nested dichotomies: an empirical analysis. Mach Learn.

[33] Beygelzimer Alina, Langford John, Lifshits Yuri, Sorkin Gregory, Strehl Alex (2009).

[34] Krzysztof Dembczyński, Wojciech Kotłowski, Willem Waegeman, Róbert Busa-Fekete, Eyke Hüllermeier. Consistency of probabilistic classifier trees. In: ECML/PKDD; 2016

[35] Frederic Morin, Yoshua Bengio. Hierarchical probabilistic neural network language model. In: Proceedings of the tenth international workshop on artificial intelligence and statistics. Society for Artificial Intelligence and Statistics; 2005. P. 246–252.

[36] Dan Hendrycks, Kevin Gimpel Gimpel. A baseline for detecting misclassified and out-of-distribution examples in neural networks. CoRR, abs/1610.02136, 2016.

[37] Shiyu Liang, Yixuan Li, Srikant R. Principled detection of out-of-distribution examples in neural networks. CoRR, abs/1706.02690, 2017.

[38] Andrey Malinin, Mark Gales. Predictive uncertainty estimation via prior networks. In: Proceedings of the 32nd international conference on neural information processing systems, NIPS’18. 2018. P. 7047–7058.

[39] Jie Ren, Peter J. Liu, Emily Fertig, Jasper Snoek, Ryan Poplin, Mark Depristo, Joshua Dillon, Balaji Lakshminarayanan. Likelihood ratios for out-of-distribution detection. In: Wallach H, Larochelle H, Beygelzimer A, d’ Alché-Buc F, Fox E, Garnett R, editors. Advances in neural information processing systems, vol. 32. Curran Associates Inc.; 2019.

[40] Joost Van Amersfoort, Lewis Smith, Yee Whye Teh, Yarin Gal. Uncertainty estimation using a single deep deterministic neural network. In: Hal Daumé III, Aarti Singh, editors. Proceedings of the 37th international conference on machine learning, Volume 119 of proceedings of machine learning research. PMLR; 2020. P. 9690–9700

[41] Kimin Lee, Kibok Lee, Honglak Lee, Jinwoo Shin. A simple unified framework for detecting out-of-distribution samples and adversarial attacks. In: Proceedings of the 32nd international conference on neural information processing systems, NIPS’18. Curran Associates Inc.; 2018. P. 7167–7177 .

[42] Chuanxing Geng Geng, Sheng-Jun Huang Huang, Songcan Chen. Recent advances in open set recognition: a survey. CoRR, abs/1811.08581, 2018. 10.1109/TPAMI.2020.2981604 32191881

[43] Raghavendra Chalapathy, Sanjay Chawla. Deep learning for anomaly detection: a survey; 2019

[44] Ethan Goan, Clinton Fookes. Bayesian neural networks: An introduction and survey. Lect Notes Math 2020;45–87.

[45] Hüllermeier Eyke, Waegeman Willem (2021). Aleatoric and epistemic uncertainty in machine learning: an introduction to concepts and methods. Mach Learn.

[46] Balaji Lakshminarayanan, Alexander Pritzel, Charles Blundell. Simple and scalable predictive uncertainty estimation using deep ensembles. In: Advances in neural information processing systems, vol. 30. Curran Associates Inc.; 2017.

[47] Murat Sensoy, Lance Kaplan, Melih Kandemir. Evidential deep learning to quantify classification uncertainty. In: Bengio S, Wallach H, Larochelle H, Grauman K, Cesa-Bianchi N, Garnett R, editors. Advances in neural information processing systems, vol. 31. Curran Associates Inc.; 2018.

[48] Bergstra James, Begio Yoshua (2012). Random search for hyper-parameter optimization. J Mach Learn Res.

[49] Pedregosa F., Varoquaux G., Gramfort A., Michel V., Thirion B., Grisel O., Blondel M., Prettenhofer P., Weiss R., Dubourg V., Vanderplas J., Passos A., Cournapeau D., Brucher M., Perrot M., Duchesnay E. (2011). Scikit-learn: machine learning in Python. J Mach Learn Res.

[50] Adam Paszke, Sam Gross, Francisco Massa, Adam Lerer, James Bradbury, Gregory Chanan et al. Pytorch: an imperative style, high-performance deep learning library. In: Wallach H, Larochelle H, Beygelzimer A, d’ Alché-Buc F, Fox E, Garnett R, editors. Advances in neural information processing systems 32. Curran Associates Inc.; 2019. P. 8024–8035.

[51] Wang Hsin-Yao, Li Wen-Chi, Huang Kai-Yao, Chung Chia-Ru, Horng Jorng-Tzong, Hsu Jen-Fu, Lu Jang-Jih, Lee Tzong-Yi (2019). Rapid classification of group b streptococcus serotypes based on matrix-assisted laser desorption ionization-time of flight mass spectrometry and machine learning techniques. BMC Bioinf.

[52] Jin Ling, Hong Wang, Gaomin Li, Zhen Feng, Yufei Song, Peng Wang, Hong Shao, Hu Zhou, Gang Chen. A novel short-term high-lactose culture approach combined with a matrix-assisted laser desorption ionization-time of flight mass spectrometry assay for differentiating escherichia coli and shigella species using artificial neural networks. PLOS One 14(10):2019;1–10. 10.1371/journal.pone.0222636 PMC6782097 31593573

[53] Esener N., Green M.J., Emes R.D., Jowett B., Davies P.L., Bradley A.J., Dottorini T. (2018). Discrimination of contagious and environmental strains of streptococcus uberis in dairy herds by means of mass spectrometry and machine-learning. Sci Rep.

[54] Rodrigues C., Passet V., Rakotondrasoa A., Brisse S. (2018). Identification of klebsiella pneumoniae, klebsiella quasipneumoniae, klebsiella variicola and related phylogroups by maldi-tof mass spectrometry. Front Microbiol.

[55] Wenhao Tang, Nisha Ranganathan, Vahid Shahrezaei, Gerald Larrouy-Maumus. Maldi-tof mass spectrometry on intact bacteria combined with a refined analysis framework allows accurate classification of mssa and mrsa. PLOS One, 14(6):2019;1–16 10.1371/journal.pone.0218951 PMC6597085 31247021

[56] Gibb Sebastian, Strimmer Korbinian (2012). MALDIquant: a versatile R package for the analysis of mass spectrometry data. Bioinformatics.

[57] Caroline Weis, Max Horn, Bastian Rieck et al. Topological and kernel-based microbial phenotype prediction from maldi-tof mass spectra. Bioinformatics 36:2020;30–38. 10.1093/bioinformatics/btaa429 PMC7355261 32657381

[58] De Bruyne Katrien, Slabbinck Bram, Waegeman Willem, Vauterin Paul, De Baets Bernard, Vandamme Peter (2011). Bacterial species identification from maldi-tof mass spectra through data analysis and machine learning. Systemat Appl Microbiol.

[59] Kévin Vervier, Pierre Mahé, Jean-Baptiste Veyrieras, Jean-Philippe Vert. Benchmark of structured machine learning methods for microbial identification from mass-spectrometry data; 2015.

[60] Fangous Marie-Sarah, Mougari Faiza, Gouriou Stephanie (2014). Classification algorithm for subspecies identification within the mycobacterium abscessus species, based on matrix-assisted laser desorption ionization-time of flight mass spectrometry. J Clin Microbiol.

[61] Sogawa Kazuyuki, Watanabe Masaharu, Ishige Takayuki (2017). Rapid staphylococcus aureus discrimination between methicillin-sensitive and methicillin-resistant using maldi-tof mass spectrometry. Biocontrol Sci.

[62] Zhuoyong Zhang, Dan Wang, Peter de B. Harrington, Kent J. Voorhees, Jon Rees. Forward selection radial basis function networks applied to bacterial classification based on maldi-tof-ms. Talanta 63:2004;527–532. 10.1016/j.talanta.2003.11.034 18969464

[63] Lasch Peter, Beyer Wolfgang, Nattermann Herbert, Stämmler Maren (2009). Identification of bacillus anthracis by using matrix-assisted laser desorption ionization-time of flight mass spectrometry and artificial neural networks. Appl Environ Microbiol.

[64] Zewen Li, Wenjie Yang, Shouheng Peng, Fan Liu. A survey of convolutional neural networks: Anal Appl Prospects CoRR, abs/2004.02806, 2020. 10.1109/TNNLS.2021.3084827 34111009

[65] Zimek Arthur, Buchwald Fabian, Frank Eibe, Kramer Stefan (2010). A study of hierarchical and flat classification of proteins. IEEE/ACM Trans Comput Biol Bioinf.

[66] Eisner Roman, Poulin Brett, Szafron Duane (2005). Improving protein function prediction using the hierarchical structure of the gene ontology. IEEE Symp Comput Intell Bioinf Comput Biol.

[67] Svetlana Kiritchenko, Stan Matwin, Fazel Famili A. Functional annotation of genes using hierarchical text categorization. NRC Publications Archive (NPArC); 2005.

[68] Sokolov Artem, Ben-Hur Asa (2010). Hierarchical classification of gene ontology terms using the gostruct method. J Bioninf Comput Biol.

[69] Fan Jianping, Zhou Ning, Peng Jinye, Gao Ling (2015). Hierarchical learning of tree classifiers for large-scale plant species identification. IEEE Trans Image Process.

[70] Carlos Silla. Hierarchical classification of bird species using their audio recorded songs. IEEE International Conference on Systems, Man, and Cybernetics; 2013.

[71] Udelhoven Thomas, Naumann Dieter, Schmitt Jürgen (2000). Development of a hierarchical classification system with artificial neural networks and ft-ir spectra for the identification of bacteria. SAGE J Appl Spectrosc.

[72] Tafintseva Valeria, Vigneau Evelyne, Shapaval Volha, Carlou Véronique (2017). Hierarchical classification of microorganisms based on high-dimensional phenotypic data. J Biophoton.

[73] Bram Slabbinck, Willem Waegeman, Peter Dawyndt, Paul De Vos, Bernard De Baets. From learning taxonomies to phylogenetic learning: integration of 16s rrna gene data into fame-based bacterial classification. BMC Bioinf 11:2010. 10.1186/1471-2105-11-69 PMC2828439 20113515

[74] Wang Q., Garrity G.M., Tiedje J.M., Cole J.R. (2007). Naive bayesian classifier for rapid assignment of rrna sequences into the new bacterial taxonomy. Appl Environ Microbol.

[75] Tz-Ying Wu, Pedro Morgado, Pei Wang, Chih-Hui Ho, Nuno Vasconcelos. Solving long-tailed recognition with deep realistic taxonomic classifier; 2020.

[76] Thomas Mortier, Marek Wydmuch, Krzysztof Dembczyński, Eyke Hüllermeier, Willem Waegeman. Efficient set-valued prediction in multi-class classification. Data Min Knowl Discovery 35:2021;1435–1469.

[77] Fangous M.-S., Mougari F., Gouriou S., Calvez E., Raskine L., Cambau E., Payan C., Héry-Arnaud G. (2014). Classification algorithm for subspecies identification within the mycobacterium abscessus species, based on matrix-assisted laser desorption ionization-time of flight mass spectrometry. J Clin Microbiol.

[78] Wang H.-Y., Li W.-C., Huang K.-Y., Chung C.-R., Horng J.-T., Hsu J.-F., Lu J.-J., Lee T.-Y. (2019). Rapid classification of group b streptococcus serotypes based on matrix-assisted laser desorption ionization-time of flight mass spectrometry and machine learning techniques. BMC Bioinf.

[79] Oberle M., Wohlwend N., Jonas D., Maurer F.P., Jost G., Tschudin-Sutter S., Vranckx K., Egli A. (2016). The technical and biological reproducibility of matrix-assisted laser desorption ionization-time of flight mass spectrometry (maldi-tof ms) based typing: employment of bioinformatics in a multicenter study. PLoS One.

[80] Geng Chuanxing, Huang Sheng-Jun, Chen Songcan (2020). IEEE Trans Pattern Anal Mach Intell.

[81] Ryne Roady, Tyler L. Hayes, Ronald Kemker, Ayesha Gonzales, Christopher Kanan. Are open set classification methods effective on large-scale datasets? PLOS One 15(9):2020;1–18 10.1371/journal.pone.0238302 PMC7473573 32886692

[82] Ian J. Goodfellow, Jonathon Shlens, Christian Szegedy. Explaining and harnessing adversarial examples. In: Yoshua Bengio, Yann LeCun, editors. 3rd International conference on learning representations, ICLR 2015, San Diego, CA, USA, May 7–9, 2015, Conference Track Proceedings; 2015.

[83] Dario Amodei, Chris Olah, Jacob Steinhardt, Paul F. Christiano, John Schulman, Dan Mané. Concrete problems in AI safety. CoRR, abs/1606.06565, 2016.

[84] Christian Szegedy, Wojciech Zaremba, Ilya Sutskever, Joan Bruna, Dumitru Erhan, Ian Goodfellow, Rob Fergus. Intriguing properties of neural networks. In: International conference on learning representations; 2014.

[85] Krzywinski M., Altman N. (2013). Points of significance: importance of being uncertain. Nat Methods.

[86] Ghahramani Zoubin (2015). Probabilistic machine learning and artificial intelligence. Natue.

[87] Stefan Depeweg, Jose-Miguel Hernandez-Lobato, Finale Doshi-Velez, Steffen Udluft. Decomposition of uncertainty in Bayesian deep learning for efficient and risk-sensitive learning. In: Proceedings of the 35th international conference on machine learning, Volume 80 of proceedings of machine learning research. PMLR, 2018. P. 1184–1193.

[88] Aryan Mobiny, Hien V. Nguyen, Supratik Moulik, Naveen Garg, Carol C. Wu. Dropconnect is effective in modeling uncertainty of bayesian deep networks; 2019. 10.1038/s41598-021-84854-x PMC7943811 33750847

[89] Yotam Hechtlinger, Barnabás Póczos, Wasserman. Cautious deep learning; 2019.

[90] Polina Kirichenko, Pavel Izmailov, Andrew Gordon Wilson. Why normalizing flows fail to detect out-of-distribution data; 2020

[91] Zhisheng Xiao, Qing Yan, Yali Amit. Likelihood regret: An out-of-distribution detection score for variational auto-encoder. In: Larochelle H, Ranzato M, Hadsell R, Balcan MF, Lin H, editors. Advances in neural information processing systems, vol. 33. Curran Associates Inc.; 2020. P. 20685–20696.

[92] Eric Nalisnick, Akihiro Matsukawa, Yee Whye Teh, Dilan Gorur, Balaji Lakshminarayanan. Do deep generative models know what they don’t know? In International Conference on Learning Representations; 2019.

[93] Martin Mundt, Iuliia Pliushch, Sagnik Majumder, Visvanathan Ramesh. Open set recognition through deep neural network uncertainty: does out-of-distribution detection require generative classifiers? In Proceedings of the IEEE/CVF International Conference on Computer Vision (ICCV) Workshops; Oct 2019.

[94] Abhijit Bendale, Terrance Boult. Towards open set deep networks. In: Computer vision and pattern recognition (CVPR), IEEE conference; 06 2016. P. 1563–1572.

[95] Mather Cheryl A., Werth Brian J., Sivagnanam Shobini, Sen Dhruba J., Butler-Wu Susan M., Burnham C.A. (2016). Rapid detection of vancomycin-intermediate staphylococcus aureus by matrix-assisted laser desorption ionization–time of flight mass spectrometry. J Clin Microbiol.

