# Supplementary material for: Bacterial species identification using MALDI-TOF mass spectrometry and machine learning techniques: A large-scale benchmarking study
Source: Comput Struct Biotechnol J. 2021 Nov 9;19:6157–68. doi: 10.1016/j.csbj.2021.11.004 (PMC8649224; doi:10.1016/j.csbj.2021.11.004)
Supplement: Supplementary file 1 [file mmc1.pdf]

6 Supplementary materials I

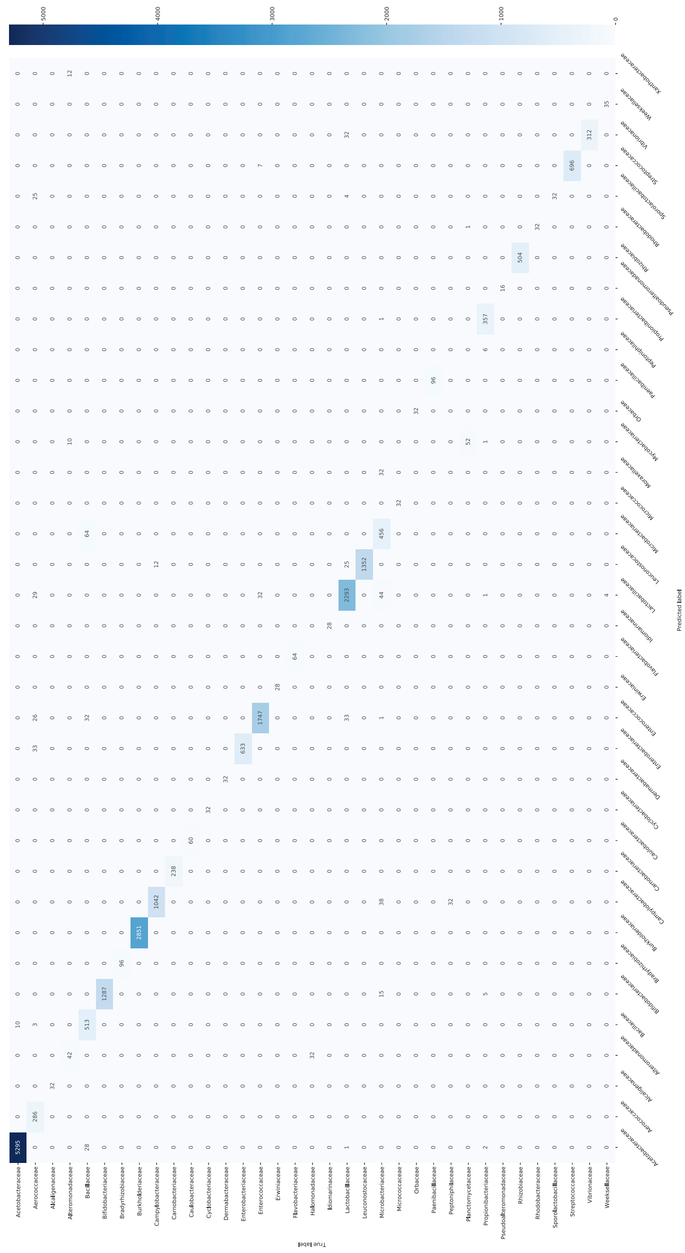

Figure 1: Confusion matrix on family level for flat KNN obtained in the novel strains scenario. We observed confusion for families *Aerococcaceae*, *Bacilaceae*, *Lactobacillaceae* and *Microbacteriaceae*.

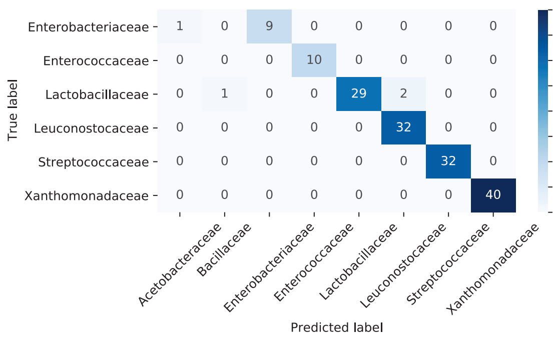

(a) Flat LSVC

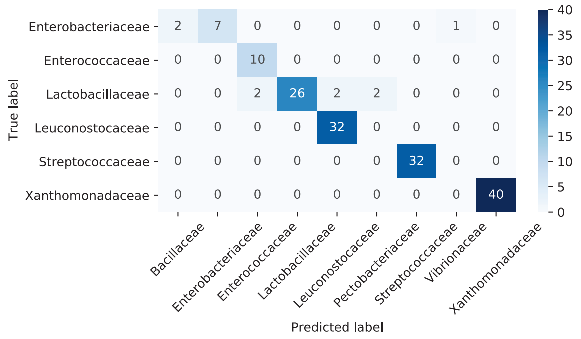

(b) Hierarchical LSVC

Figure 2: Confusion matrices on family level for LSVC obtained in the novel biological replicates scenario. Similar as in the novel strains scenario, we observed confusion for *Lactobacillaceae*.

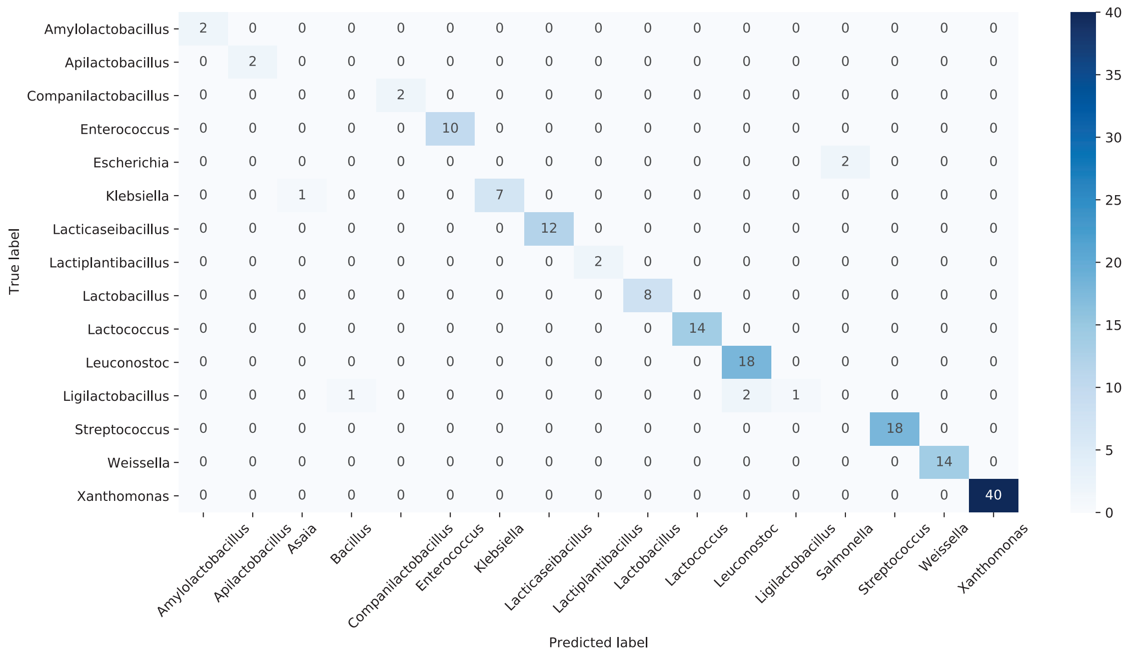

(a) Flat LSVC

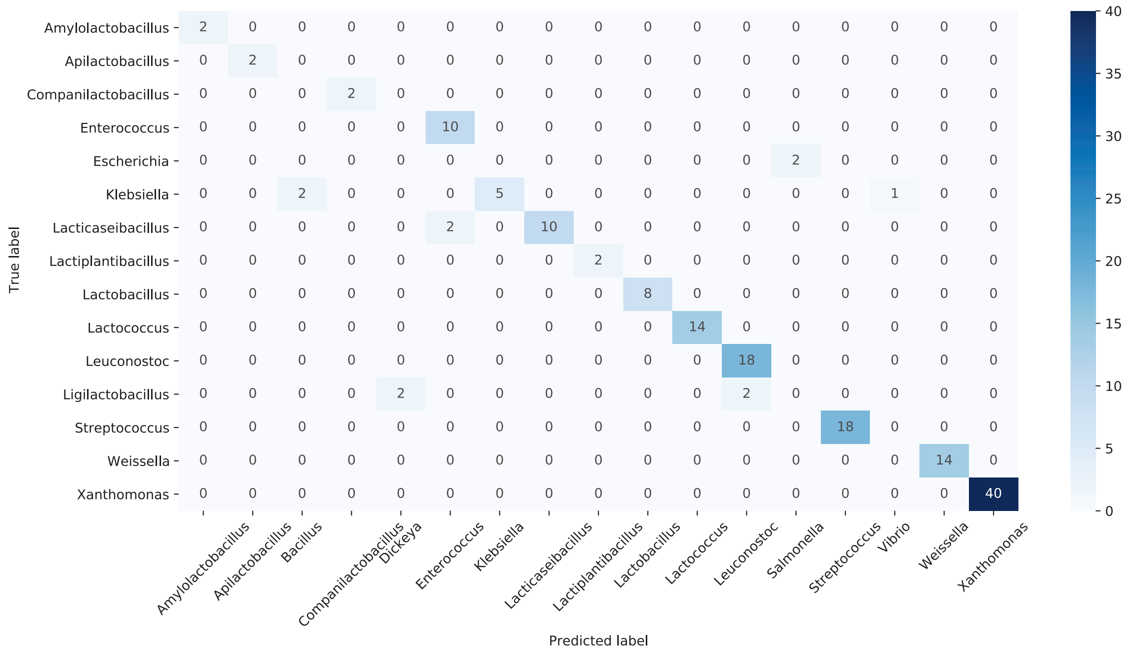

(b) Hierarchical LSVC

Figure 3: Confusion matrices on genus level for LSVC obtained in the novel biological replicates scenario.

Table 6: Overview of the different scenarios considered in the additional experiments, together with corresponding datasets. Statistics for the datasets are shown in Table 7.

SCENARIO	TRAIN	TEST
NOVEL STRAINS	GD_{train2}	$GD_{test2,ID}$
NOVEL SPECIES	GD_{train2}	$GD_{test2,ID} \cup GD_{test2,OOD}$

Table 7: Summary statistics for the different datasets used in the additional experiments (N – number of spectra, K_f – number of unique families, K_g – number of unique genera, $K_{sp.}$ – number of unique species, $K_{st.}$ – number of unique strains, ID – in-distribution, OOD – out-of-distribution).

DATASET	N	K_f	K_g	$K_{sp.}$	$K_{st.}$
GD_{train2}	79338	91	240	1035	2131
$GD_{test2,ID}$	147	22	41	110	139
$GD_{test2,OOD}$	53	24	34	52	52

7 Supplementary materials II

As an additional experiment, we compared different classifiers, excluding logistic regression due to low performance in our work, with the ClinProTool of Bruker Daltonik GmbH & Co. KG (Germany, http://www.bruker.com/) for the novel strains and novel species scenario. Moreover, we constructed a test set (GD_{test2}) by taking a random sample of 200 spectra from the global dataset. Subsequently, for the remaining part of the global dataset, we constructed a training set (GD_{train2}) by removing strains that are also present in the test set. Furthermore, the test set consists of two mutually exclusive sets: one which includes species observed during training ($GD_{test2,ID}$) and one which includes species that were not observed during training ($GD_{test2,OOD}$), respectively. The former test set is used to evaluate the novel strain identification scenario, whereas the union of former and latter is used to evaluate the novel species identification scenario – see Table 6 for an overview. Summary statistics for the different datasets are presented in Table 7.

For the Bruker software, we classify a spectrum by predicting the label of the top-1, in terms of log-score (i.e., similarity score), spectrum in the training set. When it comes to the novel species scenario, the reciprocals of the log-scores obtained by Bruker are used in the same spirit as the total uncertainty in Eqn. 5 is used by KNN and 1DQNN. When a sample from an unobserved species is analyzed by the Bruker software, one might expect a lower similarity score with the training set. Results obtained for the different scenarios are presented in Table 8 and Table 9. For both scenarios, it is clear that the Bruker software is outperformed by most of the machine learning models discussed in this work.

Table 8: Additional results for novel strains scenario. Accuracies are reported on phylogenetic levels: (F)amily, (G)enus and (S)pecies. For each machine learning model, we report the performance for flat and hierarchical classification. H-XXX denotes the hierarchical classification implementation of method XXX and Bruker denotes the ClinProTool of Bruker Daltonik GmbH & Co. KG (Germany, http://www.bruker.com/).

MODEL	Acc_F	Acc_G	Acc_S
LSVC	0.9184	0.8980	0.8027
H-LSVC	0.9388	0.9184	0.8095
RF	0.8639	0.8435	0.7211
H-RF	0.9116	0.8980	0.7551
KNN	0.9388	0.9320	0.8095
H-KNN	0.9388	0.9320	0.8095
1DCNN	0.9184	0.8980	0.7347
H-1DCNN	0.9252	0.9116	0.7551
Bruker	0.9252	0.8639	0.7551

Table 9: Additional results for novel species scenario. Area under the ROC curve (AUROC) and area under the precision-recall curve (AUPR) are reported for out-of-distribution detection based on total uncertainty for KNN and 1DCNN, and the reciprocal of the top log-score obtained by the ClinProTool of Bruker Daltonik GmbH & Co. KG (Germany, http://www.bruker.com/) for Bruker.

MODEL	AUROC	AUPR
KNN	0.6024	0.5165
1DCNN(0.2)	0.8634	0.8634
1DCNN(0.4)	0.8647	0.6259
1DCNN(0.6)	0.8588	0.6340
1DCNN(0.8)	0.8734	0.6827
Bruker	0.8312	0.5065

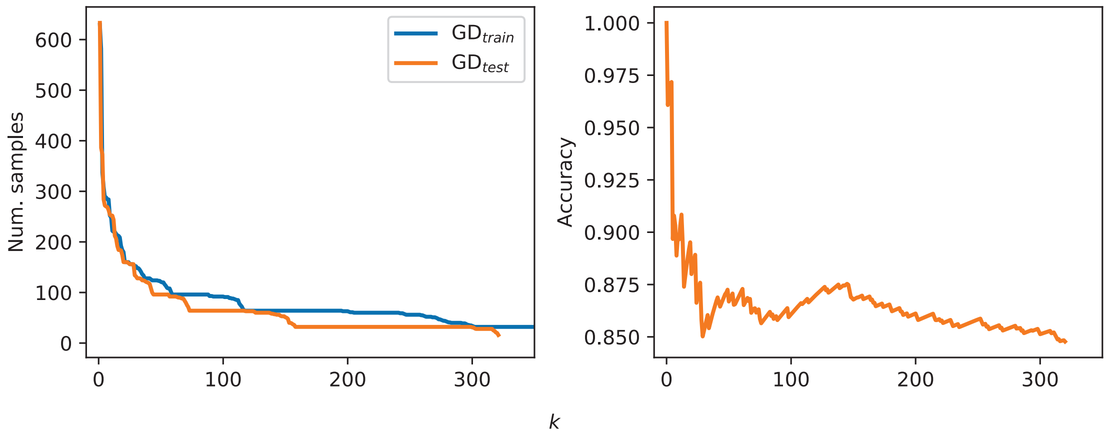

Figure 4: Left plot: number of samples (y-axis) plotted for the k most frequently observed species (x-axis) in the train (GD$_{\text{train}}$, blue) and test set (GD$_{\text{test}}$, orange) for the novel strains scenario (see Table 1). Right plot: accuracy obtained for KNN in the novel strains scenario (y-axis) when considering the k most frequent observed species (x-axis) in the test set (GD$_{\text{test}}$). A maximum accuracy is obtained when only looking at the most frequent species (i.e., $k = 1$), while the lowest accuracy is obtained when including all species in the test set (i.e., $k = 321$). From this plot, it is clear that the performance for the novel strains scenario increases when excluding the least represented species from the analysis.
